# Determining the Impact of the Reversal of Roe v. Wade on Birth Control and Infertility Trends Using Google Trends

**DOI:** 10.7759/cureus.74503

**Published:** 2024-11-26

**Authors:** Niki Parikh, Jayson Kemble, Avery Dutcher, Milad Bonakdarhashemi, Matt Ziggleman, Tobias Kohler, Sevann Helo

**Affiliations:** 1 Urology, Baylor College of Medicine, Houston, USA; 2 Urology, University of Nebraska Medical Center, Omaha, USA; 3 Urology, Ochsner Health, New Orleans, USA; 4 Urology, Mayo Clinic, Rochester, USA

**Keywords:** birth control, google, infertility, roe v. wade, trends

## Abstract

Introduction

Overturning Roe v. Wade left many concerned about birth control options and future fertility. This study aims to report Google (Google, Inc., Mountain View, CA) search trends regarding birth control and infertility options before and after the Dobb v. Jackson decision.

Methods

Google Trends (Google, Inc., Mountain View, CA) data were analyzed between January 2020 and August 2022. The relative search volume (RSV), a measure of proportional search volume, was obtained for terms related to birth control and infertility treatments. Abortion laws and median household income were compared to search term popularity. Linear regression and t-test analysis were performed.

Results

Interest in surgical birth control increased most in June 2022 compared to June 2021, with vasectomy interest increasing by 142% and tubal ligation by 98%. Surrounding the Roe v. Wade announcement, increases were seen in searches for vasectomy (90%), vasectomy reversal (142%), oral contraceptive pill (56%), plan B (36%), and in vitro fertilization (29%) (P < 0.05). Lower median household income was associated with increased interest in “vasectomy,” “tubal ligation,” and “plan B,” while higher median household income was associated with increased interest in “in vitro fertilization” (P < 0.05). Seven of the top 10 states with the highest interest in surgical birth control had laws banning abortion at any gestational age.

Conclusions

Changes in access to abortion have led to increased online interest in birth control and fertility treatment options. Surgical birth control searches were highest in states with stricter abortion laws, warranting investigation into access to reversible contraception, while the rise in fertility searches brought to light concerns regarding future laws impacting fertility care.

## Introduction

On June 24, 2022, the United States Supreme Court ruled on the Dobb v. Jackson decision, which overturned Roe v. Wade, a ruling that had previously affirmed a woman’s right to a legal abortion. The immediate effect was a ban on abortion in 13 states with automatic trigger laws that took effect [1]. Now, women across the United States have been faced with limited access to reproductive health care. A ban on abortion has far-reaching implications for men and women. It not only affects access to contraception but may also influence an individual’s decision to pursue fertility treatment options with effects on embryo storage. With the rates of ectopic pregnancy-related deaths being 5-10%, this law could have catastrophic implications regarding the management of ectopic pregnancy [2-4].

With the overturn of Roe v. Wade, there is also increased concern that future legislation may target other contraceptive methods as well as fertility treatments. We hypothesized that this would lead to increased public interest in both contraception and fertility treatment options. To gauge public interest in birth control and infertility treatments, we sought to analyze Google (Google, Inc., Mountain View, CA) search trends to determine if interest in contraception and infertility options has changed significantly since the repeal of Roe v. Wade. We present this article in accordance with the Strengthening the Reporting of Observational Studies in Epidemiology (STROBE) reporting checklist.

## Materials and methods

Google Trends (Google, Inc., Mountain View, CA) was queried to record the relative search volume (RSV) values from January 2020 to August 2022 for terms related to birth control. RSV represents a proportional interest in a search term, with values ranging from 0 to 100. A value of 50 means that a search term is half as popular at that time point compared to its peak popularity.

The following search terms were queried for interest in the United States: Roe v. Wade, abortion, vasectomy, vasectomy reversal, vasectomy cost, tubal ligation, tubal ligation reversal, tubal reversal, intrauterine device (IUD), birth control, birth control implant, oral contraceptive pill, plan B, in vitro fertilization (IVF), and testicular sperm extraction. Google Trends allows for multiple different searches of the same word, with searches grouped as a “topic” or as an exact search term. When available, the topic’s option was utilized as the topic includes searches for the search term in multiple languages. The monthly average RSVs for each search term were compared to the previous year’s monthly average, with two-month data analyzed for the timeframe surrounding the Roe v. Wade announcement. Search term popularity based on geographical location was also recorded, along with state racial breakdown and state abortion laws following the repeal of Roe v. Wade [5,6]. The provided geographical data were scaled to the state population, so state popularity reflected refers to proportional interest in each state and not the overall number of searches. The median household incomes, obtained from the United States Census, for the most and least interested 10 states for each topic were analyzed [5]. As these data are publicly available, institutional review board approval was not required.

All statistical analyses were conducted using SPSS software (version 28.0.0, IBM Corp., Armonk, NY). Descriptive statistics, linear regression, and correlation (r) analyses were performed to evaluate the relationships between variables. To explore the trends in each topic search, two metrics were calculated: (1) in each calendar year, the RSV trends throughout the 12 months were explored by linear regression analysis; (2) the RSV change for each month of 2021 and 2022 compared to the same month in the previous year was calculated using linear regression and plotted for visual comparison. Eventually, the RSV trends in different geographical regions and the household income of the highest-trend states and the lowest-trend states were compared by the Kruskal-Wallis test for each search topic. A comparison of the two months around the Supreme Court versus the year prior was performed by applying the t-test between RSV values at two time points. A two-sided P-value of less than 0.05 with a 95% confidence interval was considered significant. 

## Results

Yearly trends

Throughout 2020, when each week was compared to the same week in the prior year, only “oral contraceptive pill” had a significant decremental interest (P < 0.001, r = -0.84). In 2021, analyses showed a significant decrease in interest in “birth control” and “birth control implant” (P < 0.001, r = -0.71, and P < 0.001, r = -0.76, respectively). Furthermore, most birth control topics increased in the spring of 2021 relative to the year prior, and when compared with April 2021 and April 2020, both “vasectomy” and “vasectomy reversal” had an incremental interest of 72% (Table 1).

**Table 1:** Average monthly percent change in trends compared to average monthly interest of the previous year BC: birth control; IUD: intrauterine device; OC: oral contraceptive

	Vasectomy	Vasectomy Reversal	Tubal Ligation	IUD	BC	OC Pill	Plan B
Jan 2021	-0.9	7.5	-14.7	1.6	-3.4	-32.3	0.1
Feb 2021	-8.2	22	-8.0	3.1	6.3	-28.3	5.9
Mar 2021	52.9	79.8	26.0	24.5	15.2	-29.8	15.8
Apr 2021	72.2	72.2	31.1	32.6	18.8	-26.5	33.3
May 2021	34.4	29.3	15.3	17.3	7.1	-18.9	22.9
Jun 2021	16.8	9.8	-5.5	11.2	-3.3	-25.7	13.3
Jul 2021	13.5	4.0	-6.1	-1.4	-14.3	-29.0	12.9
Aug 2021	29.3	40	-4.6	1.9	-4.8	-21.6	6.8
Sep 2021	56.1	136.3	-5.0	4.6	-7.4	1.1	14.0
Oct 2021	-5.6	-27.8	-13.7	-0.07	-11.5	15.3	5.8
Nov 2021	11.5	19.6	-6.4	1.3	0.5	41.7	22.3
Dec 2021	10.2	-7.2	-11.5	8.2	-3.8	32.0	10.3
Jan 2022	14.6	0	0.6	2.0	-6.8	11.9	17.0
Feb 2022	3.3	-4.9	-2.3	0.3	-5.9	10.7	-3.9
Mar2022	11.8	10.2	3.4	3.1	20.9	16.1	2.5
Apr 2022	7.5	-14.5	0.8	-2.3	0.0	15.1	-0.6
May 2022	49.6	78.7	21.5	18.9	14.6	35.2	27.3
Jun 2022	142.2	226.8	97.5	12.9	47.1	84.0	47.5
Jul 2022	54.8	74.1	23.3	9.8	21.8	12.3	0.98
Aug 2022	17.6	10.4	10.9	14.9	4.9	-2.7	7.84

**Figure 1:**
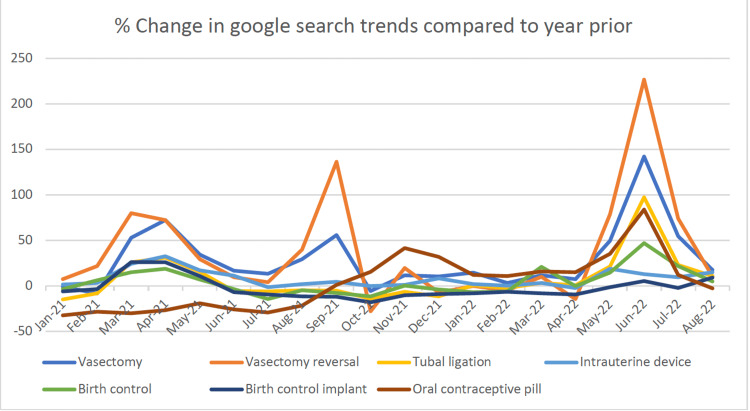
Average monthly percent change in trends compared to average monthly interest of the previous year

In 2022, most search terms increased in popularity; their peak happened in May and June, coinciding with the official announcement and leak of the Supreme Court's Roe v. Wade decision (Table 1). Interest in surgical birth control options increased the most in June 2022 compared to June 2021, with vasectomy interest increasing by 142% and tubal ligation by 98%. Interestingly, interest in vasectomy reversal also increased by 227%. Combining the two months surrounding the Roe v. Wade announcement and comparing with the previous year, significant increases were seen in searches for vasectomy (P = 0.02), vasectomy reversal (P = 0.04), oral contraceptive pill (P= 0.006), plan B (P = 0.04), and IVF (P = 0.005) (Figure 1).

**Figure 2:**
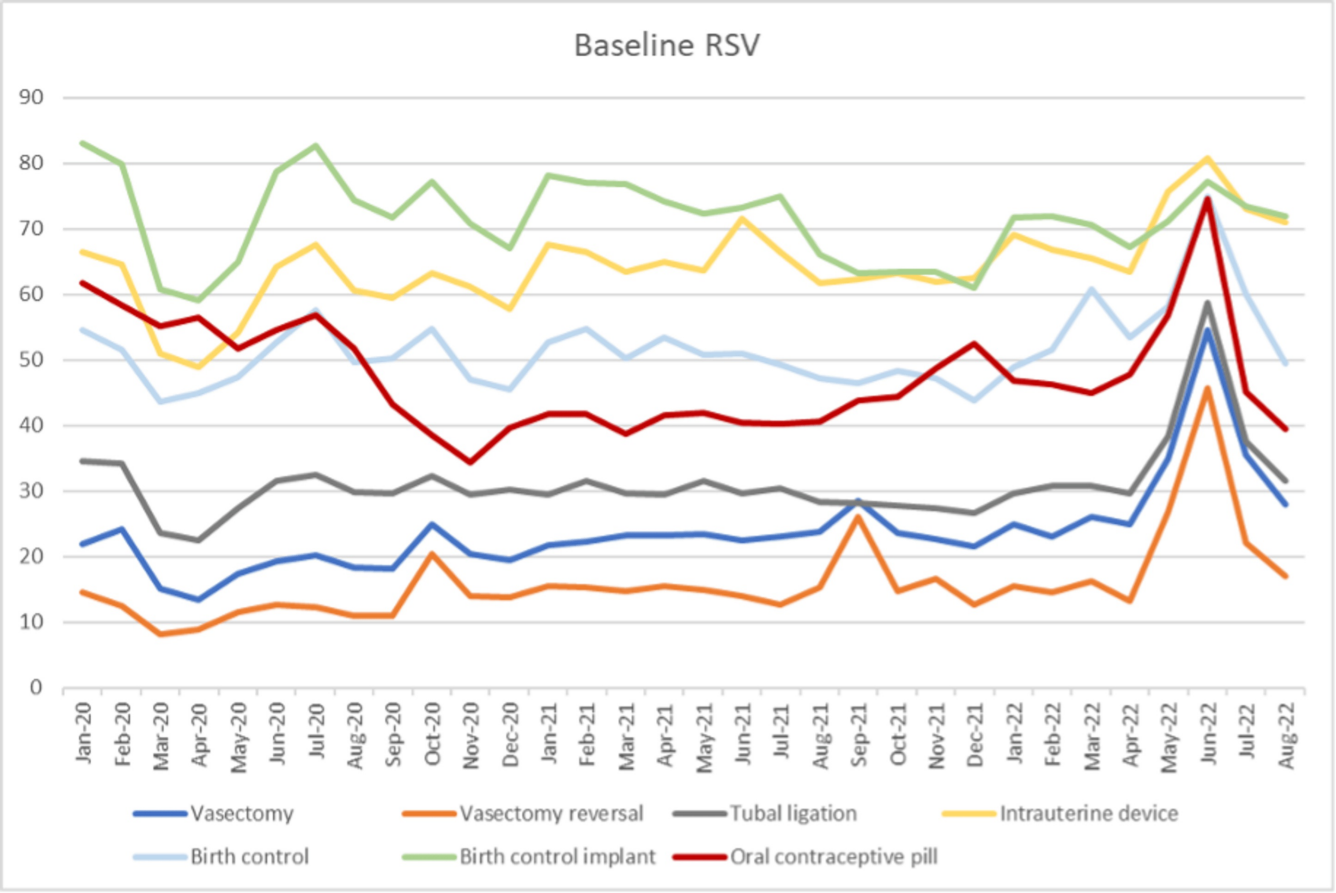
Baseline percent change in relative search volumes of search terms from January 2020 to August 2022

Trends were also evaluated between 2020 and 2022; interest in IUD and birth control decreased by 20% from March to May 2020 but quickly rebounded to normal levels (Figure 2). The analyses revealed no statistically significant changes in baseline interest for all search terms throughout this period.

Geography trends

Trends in searches based on geography were analyzed from January 2020 to August 2022 and from April 2022 to August 2022. Searches for “vasectomy” and “vasectomy reversal” were proportionally highest in the Western United States (Utah, Idaho, and Alaska) and lowest in the Northeastern United States (New York, New Jersey, and Connecticut) in both time periods. Selected search topics for April through August 2022 are highlighted in Table 2.

**Table 2:** RSV values by state from April 2022 to August 2022 RSV: relative search volume

State	Highest RSV	State	Lowest RSV
Vasectomy			
Utah	100	New York	39
Alaska	88	New Jersey	45
Oklahoma	84	Washington, DC	45
Idaho	79	Massachusetts	48
South Dakota	77	Connecticut	50
Vasectomy reversal			
Alaska	100	Connecticut	12
Utah	59	Delaware	17
Idaho	58	New Jersey	25
Oklahoma	53	West Virginia	25
Washington, DC	51	Kentucky	26
Tubal ligation			
West Virginia	100	Washington, DC	17
Arkansas	93	Hawaii	27
Oklahoma	90	New Jersey	28
Mississippi	88	New York	28
Kentucky	86	Massachusetts	30
Intrauterine device			
Utah	100	Hawaii	47
Vermont	88	New Jersey	47
Wyoming	83	Mississippi	50
Idaho	83	Pennsylvania	50
South Dakota	82	New York	51
Oral contraceptive pill			
South Dakota	100	New Mexico	22
Maine	87	Delaware	30
Wyoming	78	Idaho	35
Nevada	77	New Hampshire	37
Nebraska	73	Washington	38
In vitro fertilization			
Washington, DC	100	Vermont	29
Massachusetts	75	Wyoming	36
New Jersey	73	New Mexico	36
Delaware	69	Montana	38
New York	68	Arkansas	39
Plan B			
Louisiana	100	Maine	36
Connecticut	97	Vermont	40
Texas	85	Montana	40
Tennessee	79	New Hampshire	42
Alabama	76	Oregon	43

Between April and August 2020, the 10 states with the most interest in "vasectomy" or "vasectomy cost" had median household incomes of $50,700 and $49,790, respectively, compared to $65,190 for the 10 states with the lowest interest. States with the highest interest in “tubal ligation” had a median household income of $44,390, compared to $69,040 for the lowest interested states. In comparison, a higher median household income was associated with an increased interest in “in vitro fertilization,” at $69,040 compared to $49,060 (P < 0.05). The 10 states with the highest interest in the search term “plan B” had significantly lower median household incomes ($46,049 vs. $58,119) than the 10 states with the states with the lowest interest. Seven of the 10 states with the highest interest were in the southern US.

In the top 10 states with the highest interest in surgical birth control options, 7/10 had laws banning abortion at any gestational age, while the lowest interested states all allowed abortion until fetal viability or 24 weeks gestation, with three states having no limit on abortions. There was no significant difference in racial breakdown between states with the highest and lowest interest in surgical birth control options.

Results for “testicular sperm extraction” only had data for less than half of the United States, so it was not included in the geographical analysis. Reviewing the same Google Trends dates at a later time period occasionally had variability in the reported RSV numbers, but overall state trends generally remained the same.

## Discussion

The Google Trends search results showed an increased online interest in both birth control and infertility options following both the leak of Justice Alito’s draft opinion and the reversal of Roe v. Wade [7]. The additional peaks around March 2021 and September 2021 appear to correspond to the months immediately prior to the U.S. Supreme Court’s decision to hear the Dobb v. Jackson case on May 17, 2021, and the first oral arguments on December 1, 2021 [8]. This suggests that the increased online interest may be a direct result of the Supreme Court’s ruling. When analyzing birth control options, surgical birth control searches “tubal ligation” and “vasectomy” increased significantly. Interestingly, states with the highest interest in surgical birth control had some of the strictest abortion laws and lower median household incomes compared to less interested states. These data suggest that individuals most affected by the change in abortion laws may have increased interest in permanent forms of birth control.

Some factors to consider when evaluating forms of birth control include access, convenience, risks, and cost. Kavanaugh et al. examined unfulfilled contraceptive preferences and found that cost was a major barrier, accounting for unfulfilled preferences in nearly 25% of contraceptive users and 40% of nonusers [9]. In addition, racial and ethnic inequalities continue to prevail and affect fertility care in the United States, including access to contraception [10]. A study by Patel et al. discusses the lack of access to urologists. Their study found that states with the highest RSVs for “vasectomy” correlated to the lowest urologist-to-adult-male ratio. The negative effects of a shortage of urologic physicians may be further amplified as the need for urologic services increases [11].

Google Trends analysis demonstrated an increase in search interest for “plan B” in the two months surrounding Roe v Wade. The increase in search volumes corresponded to a rise in product purchases, as sales increased by 600% 24 hours after the Supreme Court announcement, and 72% of these purchases were in multiples [12]. Barriers to contraceptive purchase and use are largely influenced by racial and ethnic disparities, cost, and location [13]. In the past, higher income levels have correlated with higher rates of emergency contraceptive pill (ECP) use [14]. Interestingly, states with the highest search interest correlated with lower median household incomes. However, due to the implementation of new laws, it is possible that those with financial disparities will be more likely to purchase and use ECPs, as this may be the only cost-effective option to avoid an unwanted pregnancy. The US Department of Agriculture estimates that the cost of raising a child is roughly $300,000, and thus, this may influence those without the financial means to purchase a cheaper option, such as a $50 ECP, as a preventative measure [15].

While contraceptive options showed a significant increase in search volume, fertility-related searches “vasectomy reversal” and “in vitro fertilization” also increased. The IVF process may result in the creation of multiple embryos, of which one or two may be transferred per cycle. If successful, an individual or couple may be faced with the decision of what to do with the remaining embryos. In 1978, the first baby was born via IVF, five years after the Roe v. Wade decision. This decision may have protected IVF rights as the law specifically allowed a person to have an abortion until the fetus becomes viable [16]. Newly implemented laws now state that life begins at fertilization, and thus, a fetus is given the rights of a person. As a result, couples may be prohibited from destroying frozen embryos, and physicians may face legal challenges if embryos are destroyed or are not implanted properly [17]. In fact, the opinion written by Justice Alito mentions an “unborn human being” multiple times [7]. The rise in fertility searches may be due to a fear of laws impacting access to future fertility care. Some states (4/5) with the highest relative search values for vasectomy were also the highest for vasectomy reversal. This tandem increase in searches is likely due to individuals wanting to fully grasp the implications of undergoing a vasectomy and the future reversibility of this relatively permanent option. Unfortunately, fertility care is only the tip of the iceberg, and this decision will have future impacts on pregnancy and pregnancy complications [18].

These findings are further supported by Ghomeshi et al., whose study demonstrated similar outcomes of a significant increase in search volume interest in both “vasectomy” and “tubal ligation.” However, this study demonstrated the increase for seven days following the Supreme Court leaked draft regarding Roe v. Wade, while our study further demonstrates the trend of increased search volumes in both May and June, assessing the response to both the leaked and formal announcement [19]. Additionally, a recent study completed by Sax et al. demonstrated similar trends, specifically for the term “vasectomy” during both time periods. This study is novel as it is the only study that has analyzed infertility search terms, provided a comparison of reversible and non-reversible contraceptive options, and correlated this data to state income levels [20,21].

Limitations

While Google Trends is a valuable resource that has been increasingly utilized in public health projects, there are several limitations to this search tool. The trends are not a substitute for public interest, and Google Trends excludes those who do not have access to the internet. Other factors such as patient age, education level, media coverage, and the COVID-19 pandemic also could not be accounted for as potential confounders. Additionally, there are likely differences in search patterns between different ethnic groups and different socioeconomic groups. Google Trends has no validated content, data on algorithms used are not made available, and the context of the searches is unclear as interest in a topic may not be the only reason for a search. Lastly, the search items may also not be displayed if the search index is negligible, as shown by the results for the search term “TESE.” Additionally, because Google Trends only provides RSVs and not raw data, data analysis of Google Trends data is limited. This study also analyzed the relative search terms or changes in search term popularity over time. Therefore, Figure 1 does not allow for a comparison of search term popularity within variables.

Google Trends data collection process was updated on January 1, 2022. A study performed after this change noted some variance in extracted data, as expected values in 2022 were higher after the implemented changes compared to before the change. While these changes may have skewed the results, the changes seen in this study were significant enough to still be reflective of real-world changes [22].

While this study looks at a snapshot of search terms around the time of the Dobb v. Jackson decision and the media interest surrounding the decision, the number of patients who underwent these treatments was unable to be elucidated. Previous works, however, have confirmed these findings. Bole et al. identified a significant increase in men undergoing vasectomy consults and vasectomy consults following the reversal of Roe v. Wade [23]. This was determined by a chart review of patients as well as billing data. While the search trends may have peaked around the Roe v. Wade decisions, with long-term trends returning to pre-hearing baselines, these data do have real-world applications and long-lasting implications. Further work is needed to determine the real-world impact of these findings on infertility procedures and birth control options.

Future steps

With the uptick in searches for surgical contraception, access to and availability of all forms of contraception needs to be assessed. The same is true for facilities that provide IVF. While those with means may be able to obtain IVF by traveling to other states, the low-income, underprivileged members of the community will lose access. These results may help public health officials provide outreach to the specific counties, minorities, and underprivileged that are most impacted by this decision.

These data suggest a possible uptick in men seeking vasectomies, and we must, therefore, adjust our practices to support this increased demand. This is especially true in rural areas where a shortage of urologists is already present [24]. The impacts of Roe v. Wade on embryo creation may also impact urologists specializing in infertility as well as Reproductive Endocrinology and Infertility colleagues due to the potential lower utilization of IVF and intracytoplasmic injection. The long-term ramifications of this are yet to be determined. Changes to female fertility treatment options will likely have ripple effects for male fertility-related procedures such as vasectomy reversals and testicular sperm extraction. Such changes will affect patient care and trainee education. Future work, including longitudinal studies or demographic-focused investigations, may guide future inquiries.

## Conclusions

The enactment of laws limiting women’s access to abortion has wide-ranging implications for both men and women surrounding their fertility and reproductive care. Changes in access to abortion have led to increased online interest in birth control and infertility options. This is especially true in states with lower median incomes. The rise in contraceptive searches, especially of surgical birth control, may emphasize the lack of access to nonsurgical contraception, while the rise in fertility searches may be due to fear of laws impacting access to future care. Further studies are needed to determine if actual rates of surgical contraception and reversal of contraception rise in congruence with Google search volumes.
